# Long-Term Control of Refractory Cardiogenic Pleural Effusion in a Cat Treated with Sacubitril/Valsartan and Hydrochlorothiazide: A Case Report

**DOI:** 10.3390/ani16172701

**Published:** 2026-08-31

**Authors:** Mălina-Cristina Maftei, Laura Marina Scîntei, Sorin Ioan Beschea Chiriac, Vasile Vulpe, Radu Andrei Baisan

**Affiliations:** Faculty of Veterinary Medicine, “Ion Ionescu de la Brad” Iasi University of Life Sciences, 700490 Iasi, Romania; malina.maftei@iuls.ro (M.-C.M.); laura.bilboc@iuls.ro (L.M.S.); sorin.beschea@iuls.ro (S.I.B.C.); vasile.vulpe@iuls.ro (V.V.)

**Keywords:** nonspecific phenotype cardiomyopathy, endomyocardial fibrosis, angiotensin receptor/neprilysin inhibitor, congestive heart failure

## Abstract

Sacubitril/valsartan is an angiotensin receptor–neprilysin inhibitor (ARNI) widely used in human heart failure and has demonstrated potential cardiorenal and neurohormonal effects in experimental canine studies. Hydrochlorothiazide is a thiazide diuretic that acts at a more distal segment of the nephron than furosemide. Its addition to loop diuretic therapy may enhance sodium and fluid excretion through sequential nephron blockade and thereby improve the diuretic response in cases of refractory congestion. However, information regarding the combination of these two drugs in cats with congestive heart failure is lacking. This report describes the long-term clinical outcome of a cat with recurrent cardiogenic pleural effusion treated with sacubitril/valsartan and hydrochlorothiazide. A 14-year-old spayed female Persian-cross cat was referred for severe respiratory distress caused by recurrent pleural effusion. Echocardiography identified advanced cardiomyopathy with a nonspecific phenotype and overlapping hypertrophic and restrictive features, including focal basal septal hypertrophy, biatrial enlargement, atrial fibrillation and spontaneous echocardiographic contrast. Despite conventional treatment, which included pimobendan, torasemide, and antithrombotic medications, the cat experienced multiple episodes of pleural effusion over the following months, requiring repeated thoracocentesis. Consequently, the treatment regimen was expanded to include sacubitril/valsartan and hydrochlorothiazide. According to the owner, all other cardiac medications were discontinued approximately one week later without veterinary consultation. At long-term follow-up approximately two years after sacubitril/valsartan and hydrochlorothiazide therapy initiation, the cat remained clinically stable, with no further episodes of respiratory distress. Echocardiography revealed persistent severe structural heart disease, although improved left ventricular systolic indices were observed. This case report describes prolonged clinical stabilization in a cat with advanced cardiomyopathy and recurrent congestive heart failure following the administration of sacubitril/valsartan and hydrochlorothiazide over a two-year period. Whether this outcome reflects a specific effect of sacubitril/valsartan, the addition of hydrochlorothiazide, or a combination of both cannot be determined from a single observation. Nonetheless, the duration and completeness of the response observed here warrant prospective evaluation of combination of ARNI therapy and hydrochlorothiazide in cats with refractory congestive heart failure. Further studies are needed to assess the safety and effectiveness of this approach in feline cardiomyopathy.

## Simple Summary

Recurrent pleural effusion is a common and serious complication of advanced heart disease in cats, which can cause repeated dyspnea. The cat with severe heart disease reported in this paper experienced recurrent pleural effusion despite conventional treatment. After intervention with sacubitril/valsartan, a drug commonly used to treat human heart failure, in combination with diuretics, the cat has had no recurrence of clinical signs for two years. Although conclusions cannot be drawn from a single case, this observation suggests that sacubitril/valsartan in combination with hydrochlorothiazide may represent a promising treatment option for cats with advanced heart disease and warrants further investigation.

## 1. Introduction

Feline cardiomyopathies are the most frequent cardiac diseases in cats, which often lead to congestive heart failure (CHF) [1]. Advanced myocardial remodeling can cause severe diastolic dysfunction, atrial enlargement, pulmonary edema, and pleural effusion. Pleural effusion is a common complication of CHF in cats, resulting in recurrent breathing difficulties and repeated hospitalization [2]. Classifying feline cardiomyopathies can be challenging because of substantial overlap among phenotypic presentations and mixed remodeling patterns seen in advanced stages, in particular end-stage hypertrophic cardiomyopathy (HCM) that may evolve toward a phenotype characterized by left ventricular wall thinning and restrictive physiology, closely resembling restrictive cardiomyopathy (RCM) [3,4]. Endomyocardial forms of RCM may present various structural abnormalities such as irregular hyperechoic endocardial regions, fibrotic lesions, papillary muscle irregularities, myocardial thinning, and apical remodeling resembling an aneurysm. These changes are associated with worse outcomes in cats [5,6]. Current treatments for feline congestive heart failure mainly target congestion control and prevention of thromboembolic events [7]. However, some cats may experience recurrent or refractory CHF despite maximized conventional therapy.

Sacubitril/valsartan is a novel medication that combines valsartan, an angiotensin II receptor blocker, with sacubitril, a neprilysin inhibitor, known as an angiotensin receptor–neprilysin inhibitor (ARNI). By blocking neprilysin, the drug prevents degradation of natriuretic peptides, thereby augmenting natriuresis and vasodilation. At the same time, valsartan attenuates RAAS-mediated vasoconstriction and aldosterone secretion. The net effect is a dual modulation of neurohormonal pathways that neither drug class achieves independently [8]. In human medicine, the PARADIGM-HF trial established sacubitril/valsartan as superior to enalapril in reducing cardiovascular death and heart failure hospitalizations in patients with systolic dysfunction [9]. The PARAGON-HF trial further evaluated sacubitril/valsartan in heart failure with preserved ejection fraction, a phenotype sharing key features with restrictive feline cardiomyopathy, including diastolic dysfunction, myocardial fibrosis, and elevated filling pressures, and although the primary endpoint was not met, subgroup analyses indicated potential benefit beyond the heart failure with reduced ejection fraction spectrum [10]. Although evidence in veterinary medicine remains limited, several experimental and clinical studies in dogs have reported encouraging findings. In a canine model of RAAS activation, both valsartan and sacubitril/valsartan significantly reduced aldosterone exposure while enhancing natriuretic peptide activity through cGMP-mediated pathways, suggesting that neprilysin inhibition may provide additional suppression of aldosterone beyond that achieved with RAAS blockade alone [11]. These data led the authors to propose sacubitril/valsartan as a pharmacological candidate worth investigating in clinical canine heart disease. In dogs with naturally occurring myxomatous mitral valve disease, sacubitril/valsartan was well tolerated and reduced urinary aldosterone concentrations without adversely affecting blood urea nitrogen, serum creatinine, electrolyte concentrations, or systolic arterial pressure [12]. Experimental studies in dogs with cardiorenal syndrome demonstrated that sacubitril/valsartan improved left ventricular systolic function and mitochondrial function while decreasing biomarkers of both cardiac and renal injury [13]. Improved renal haemodynamics have also been described in healthy dogs receiving ARNI therapy [8]. Short-term administration of sacubitril/valsartan has also been associated with reverse cardiac remodeling, as reflected by favorable changes in several echocardiographic indices in dogs with symptomatic myxomatous mitral valve disease [14]. Long-term administration appears safe in dogs and median survival time was increased [15]. Taken together, these canine studies point toward consistent neurohormonal, renal, and structural benefits of ARNI therapy across multiple disease contexts.

Hydrochlorothiazide is a thiazide diuretic that acts on the distal convoluted tubule by inhibiting sodium and chloride reabsorption, resulting in mild to moderate diuresis. In patients with refractory congestive heart failure, thiazide diuretics may be used as adjunctive therapy to loop diuretics to enhance natriuresis through sequential nephron blockade, by limiting distal sodium reabsorption that persists despite loop diuretic administration [16]. Although their use in veterinary patients with congestive heart failure remains limited, hydrochlorothiazide may provide an additional diuretic effect when conventional loop diuretic therapy is insufficient to achieve adequate control of congestion [17].

To date, there are no clinical reports on long-term use of sacubitril/valsartan in cats with refractory congestive heart failure. This report aims to detail the treatment of a cat with recurrent cardiogenic pleural effusion due to advanced cardiomyopathy with a nonspecific phenotype, successfully managed with sacubitril/valsartan therapy and hydrochlorothiazide.

## 2. Case Presentation

A 14-year-old spayed female Persian-cross cat weighing 3.15 kg was referred to the Emergency Department for further evaluation of severe respiratory distress. Earlier on the day of presentation, thoracocentesis had been performed at the referring clinic, with approximately 65 mL of serosanguineous pleural fluid removed, and routine hematological and biochemical analyses had been obtained.

On presentation, the cat was laterally recumbent and exhibited severe respiratory distress characterized by tachypnea, marked inspiratory effort, paradoxical abdominal respiration, and intermittent open-mouth breathing. Rectal temperature was 36.2 °C, consistent with hypothermia secondary to poor peripheral perfusion and respiratory compromise. Respiratory rate was 125 breaths/minute. Cardiac auscultation revealed a gallop sound. As part of the initial emergency assessment, a thoracic point of care ultrasound was performed and identified a large volume of pleural fluid. Emergency thoracocentesis was performed immediately after admission because of severe respiratory compromise, resulting in removal of approximately 200 mL of pleural fluid with the partial improvement in respiratory pattern. The patient was hospitalized for oxygen supplementation, stabilization, and further diagnostic evaluation.

**Figure 1:**
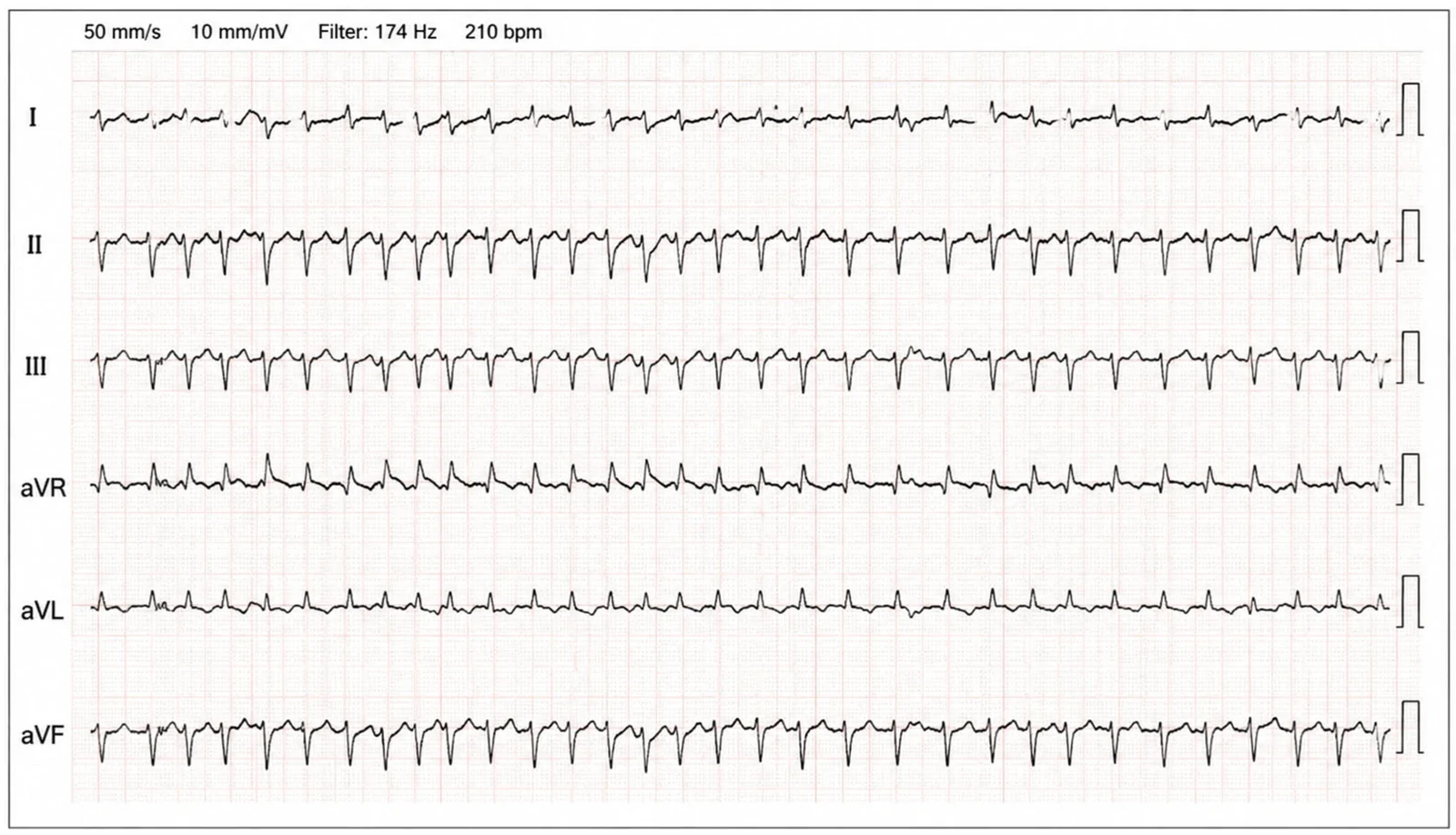
Electrocardiogram (50 mm/s; 10 mm/mV). Irregularly irregular rhythm with the absence of P waves, consistent with atrial fibrillation with a ventricular rate of 240 bpm.

Following stabilization, a complete cardiologic examination was performed. Six-lead electrocardiography (PolySpectrum 8E/8V, Ivanovo, Russia) showed an irregularly irregular supraventricular tachyarrhythmia characterized by the absence of discernible P waves, consistent with atrial fibrillation. Ventricular response rate ranged between 210 and 240 bpm. QRS complexes were narrow, with left axis deviation and deep Q-waves in leads II, III and aVF (Figure 1).

Echocardiography was performed in right and left lateral recumbence as previously described [18] using a Mindray, Vetus 8 ultrasound machine (Mindray, Animal Care, Shenzhen, China) equipped with a 4–10 MHz phased-array transducer. Most echocardiographic examinations were performed during episodes of clinical decompensation and after initial stabilization of the patient. Table 1 summarizes the data comparison between the first visit and after the introduction of sacubitril/valsartan.

**Figure 2:**
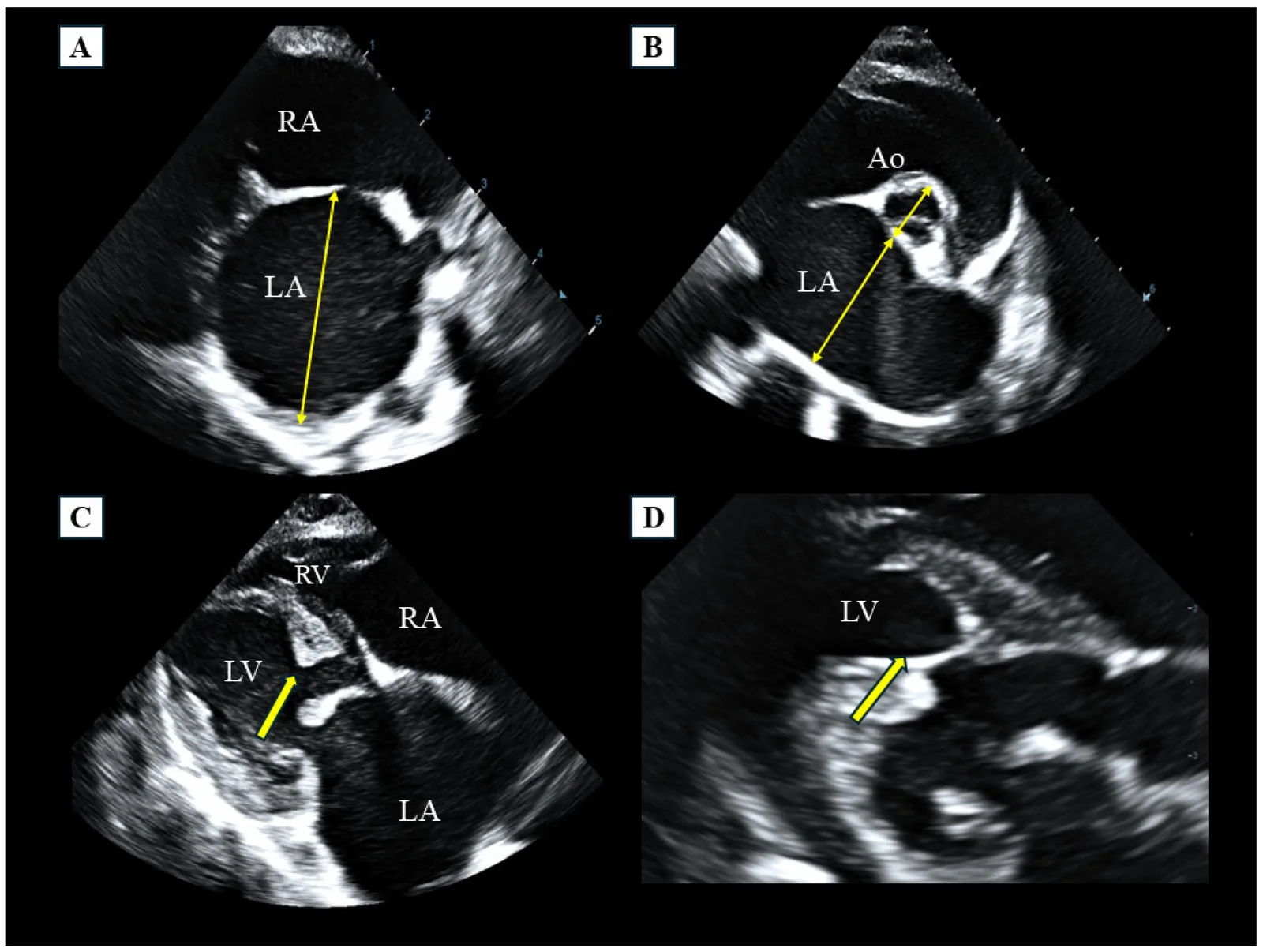
Echocardiographic phenotype of unclassified cardiomyopathy with severe biatrial enlargement. (**A**) Right parasternal four-chamber view demonstrating severe left atrial (LA) enlargement and concurrent right atrial (RA) enlargement. (**B**) Right parasternal short-axis view at the level of the aortic root showing marked left atrial enlargement with an increased LA/Ao ratio and increase left atrial appendage. (**C**) Right parasternal four-chamber view with focal basilar interventricular septal hypertrophy (arrow) within the left ventricle. (**D**) Right parasternal four-chamber view demonstrating a left ventricular band extending between a papillary muscle and the interventricular septum (arrow). Abbreviations: RA: right atrium; RV: right ventricle; LV: left ventricle; AO: aorta.

Severe left atrial enlargement was observed, with left atrial antero-posterior diameter (LAD) of 27.2 mm and a LA/Ao of 2.67 (Figure 2A,B), and with spontaneous echocardiographic contrast (SEC) in the left atrium and left auricle, indicating marked intracardiac blood stasis and advanced atrial remodeling. The right atrium was also mildly enlarged, with an anteroposterior diameter (RAD) of 12.9 mm, exceeding published reference values for healthy cats and supporting the presence of biatrial remodeling [19].

Left ventricular wall measurements showed asymmetric myocardial remodeling. Focal asymmetric thickening of the basal interventricular septum of the left ventricular outflow tract was identified (Figure 2C), characterized by increased endocardial hyperechogenicity suggestive of fibrotic remodeling with the maximum interventricular septal thickness in diastole (IVSd) measuring 6.2 mm. The left ventricular free wall thickness in diastole (LVPWd) was normal, measuring 4.4 mm. Pronounced papillary muscle hypertrophy was also observed. The heterogeneous myocardial echogenicity associated with diffuse hypokinesis and regional apical myocardial thinning may be compatible with aneurysmal-like remodeling. Additionally, prominent hyperechoic trabecular and bridging structures extending between the interventricular septum and left ventricular free wall were observed, resembling the large bridging scars and endomyocardial bands previously described in the endomyocardial form of feline restrictive cardiomyopathy. Multiple hyperechoic left ventricular bands extending between the papillary muscles and interventricular septum were also present (Figure 2D).

The mitral valve leaflets appeared mildly thickened and irregular, with color and continuous-wave Doppler interrogation demonstrating mitral regurgitation. Continuous-wave Doppler examination identified a trivial mitral regurgitant jet. Systolic anterior motion of the anterior mitral leaflet was observed, likely secondary to focal basal interventricular septal hypertrophy. Mild turbulence was detected within the left ventricular outflow tract, generating a peak systolic gradient of approximately 23 mmHg, which was considered hemodynamically non-significant.

Systolic function was at the inferior limit, with a left ventricular fractional shortening (FS) of 36.43%. Assessment of diastolic function was limited by atrial fibrillation, which precluded determination of the transmitral E/A ratio owing to the absence of an identifiable A wave. The peak transmitral E-wave velocity was 1.09 m/s.

A mild tricuspid regurgitation was detected following color Doppler interrogation. The estimated tricuspid regurgitation pressure gradient was 20.8 mmHg, which did not support the presence of clinically significant pulmonary hypertension.

Overall, the echocardiographic findings were most consistent with an advanced nonspecific cardiomyopathy phenotype. Severe left atrial enlargement, mild right atrial enlargement, spontaneous echocardiographic contrast, atrial fibrillation, congestive heart failure, and advanced myocardial remodeling were evident. The coexistence of focal basal septal hypertrophy, papillary muscle hypertrophy, and regional myocardial thinning with hypokinesis suggested an overlap between hypertrophic and restrictive cardiomyopathy phenotypes. Although the echocardiographic findings were suggestive of a nonspecific phenotype, these findings were consistent with an advanced myocardial disease.

Complete blood count and serum biochemical analyses were performed, and the findings were like those obtained at the referring clinic. No clinically relevant abnormalities apart from mild lymphopenia (0.62 × 10^9^/L; reference interval 0.83–9.1 × 10^9^/L) and a marginal increase in blood urea nitrogen concentration (32 mg/dL; reference interval 10–30 mg/dL) were identified, the latter considered consistent with the effects of furosemide administration. Serum creatinine, electrolyte concentrations, liver enzyme activities, total protein, and albumin concentrations remained within reference intervals.

Cytologic examination of the pleural fluid identified a lactescent effusion with increased triglyceride content. Total nucleated cell count was 716 cells/µL (0.72 × 10^3^/µL). Cytologic evaluation revealed a predominance of lymphocytes (>70%), with smaller populations of neutrophils (25%) and monocytes/eosinophils (5%). Based on lactescent appearance, increased triglyceride content, and lymphocyte predominance (>70%), the effusion was classified as a chylous effusion. Formal triglyceride quantification was not available for confirmatory ratio-based classification.

**Figure 3:**
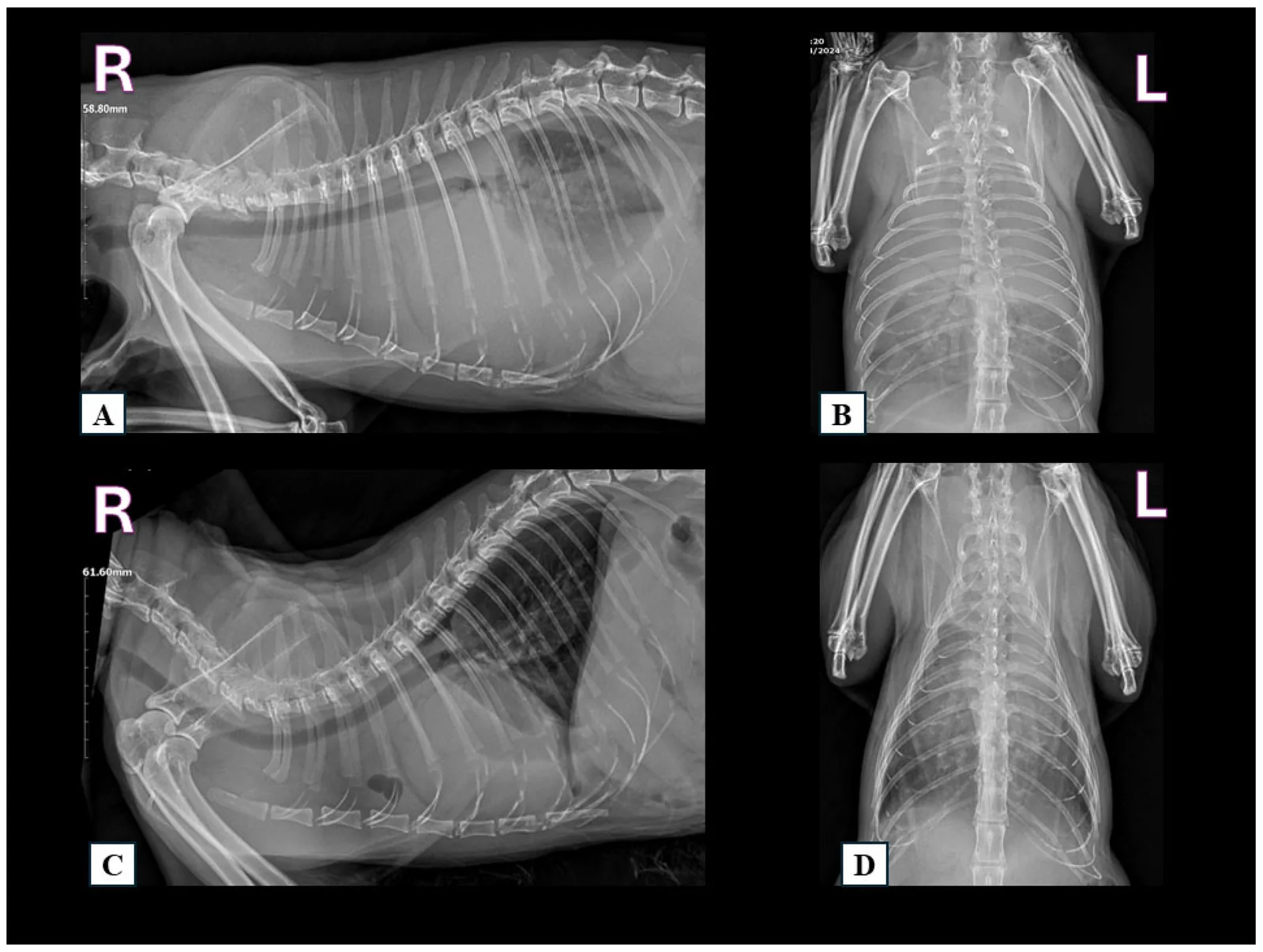
Thoracic radiographs obtained at presentation before and after thoracocentesis. (**A**,**B**) Right lateral and dorsoventral projections before pleural drainage: severe bilateral pleural effusion with lung lobe retraction and effacement of the cardiac silhouette. (**C**,**D**) Corresponding projections after removal of ~200 mL of pleural fluid: partial pulmonary re-expansion, generalised cardiomegaly, residual pleural effusion, and interstitial-to-alveolar infiltrates consistent with cardiogenic pulmonary oedema.

Thoracic radiographs were obtained using a radiographic unit (MAXIVET 400 HF, OR Medicale s.r.l., Cavaria, Italy) during episodes of respiratory distress. Radiographs acquired before thoracocentesis revealed severe pleural effusion with marked lung lobe retraction and diffuse pleural soft-tissue opacity, resulting in pronounced loss of pulmonary aeration (Figure 3A,B). Following therapeutic thoracocentesis, a marked reduction in pleural effusion and partial pulmonary re-expansion were observed. Residual findings included persistent cardiomegaly, a small volume of remaining pleural effusion, and pulmonary interstitial-to-alveolar infiltrates compatible with cardiogenic pulmonary edema (Figure 3C,D).

Following stabilization and resolution of severe respiratory distress, the cat was discharged with guideline-based medical management for congestive heart failure and thromboembolic prevention. Treatment consisted of pimobendan (0.625 mg PO q12h), clopidogrel (18.75 mg PO q24h), rivaroxaban (2.5 mg PO q24h), and torasemide (0.75 mg PO q24h for 7 days followed by 0.5 mg PO q24h) with reassessment planned one week after discharge.

Eight days after discharge, the cat was referred to the emergency service because of recurrent respiratory distress associated with re-accumulation of pleural effusion. Approximately 200 mL of pleural fluid was removed by thoracocentesis. The owners reported inconsistent administration of the prescribed diuretic therapy. Following stabilization, the patient was discharged with the same therapeutic recommendations.

Three weeks later, the cat returned with recurrence of dyspnea and pleural effusion. Therapeutic thoracocentesis yielded approximately 120 mL of pleural fluid. Owing to persistent congestive signs, the torasemide dosage was adjusted to 0.75 mg PO q24h.

The next cardiologic re-evaluation was performed approximately four months later. However, three weeks after that visit, the patient again developed respiratory distress requiring emergency thoracocentesis. Approximately 200 mL of pleural fluid and 50 mL of pericardial fluid were removed.

Two months later, another episode of recurrent congestive decompensation occurred, with approximately 200 mL of pleural effusion evacuated by thoracocentesis. Given the repeated relapses despite conventional therapy, the medical protocol was modified to include sacubitril/valsartan and hydrochlorothiazide. The cat was discharged with the following treatment regimen: pimobendan (0.625 mg PO q12h), torasemide (0.5 mg PO q12h), rivaroxaban (2.5 mg PO q24h), clopidogrel (18.75 mg PO q24h), sacubitril/valsartan (Entresto^®^, Novartis Europharm Limited, Dublin, Ireland, 24/26 mg; 1/2 tablet PO q12h), and hydrochlorothiazide (6.25 mg PO q24h).

The cat was not presented for emergency respiratory decompensation during the subsequent 2-year follow-up period, nor brought for routine cardiologic examination. According to the owner, no further episodes of clinically relevant pleural effusion or dyspnea were observed during this interval. At the time of long-term evaluation, the owner reported that approximately one week after discharge, the prescribed treatment regimen had been modified without prior veterinary consultation. Sacubitril/valsartan (Entresto^®^ 24/26 mg) was subsequently administered at a dosage of one tablet PO q24h, hydrochlorothiazide remained the same, but all other medications were discontinued.

**Figure 4:**
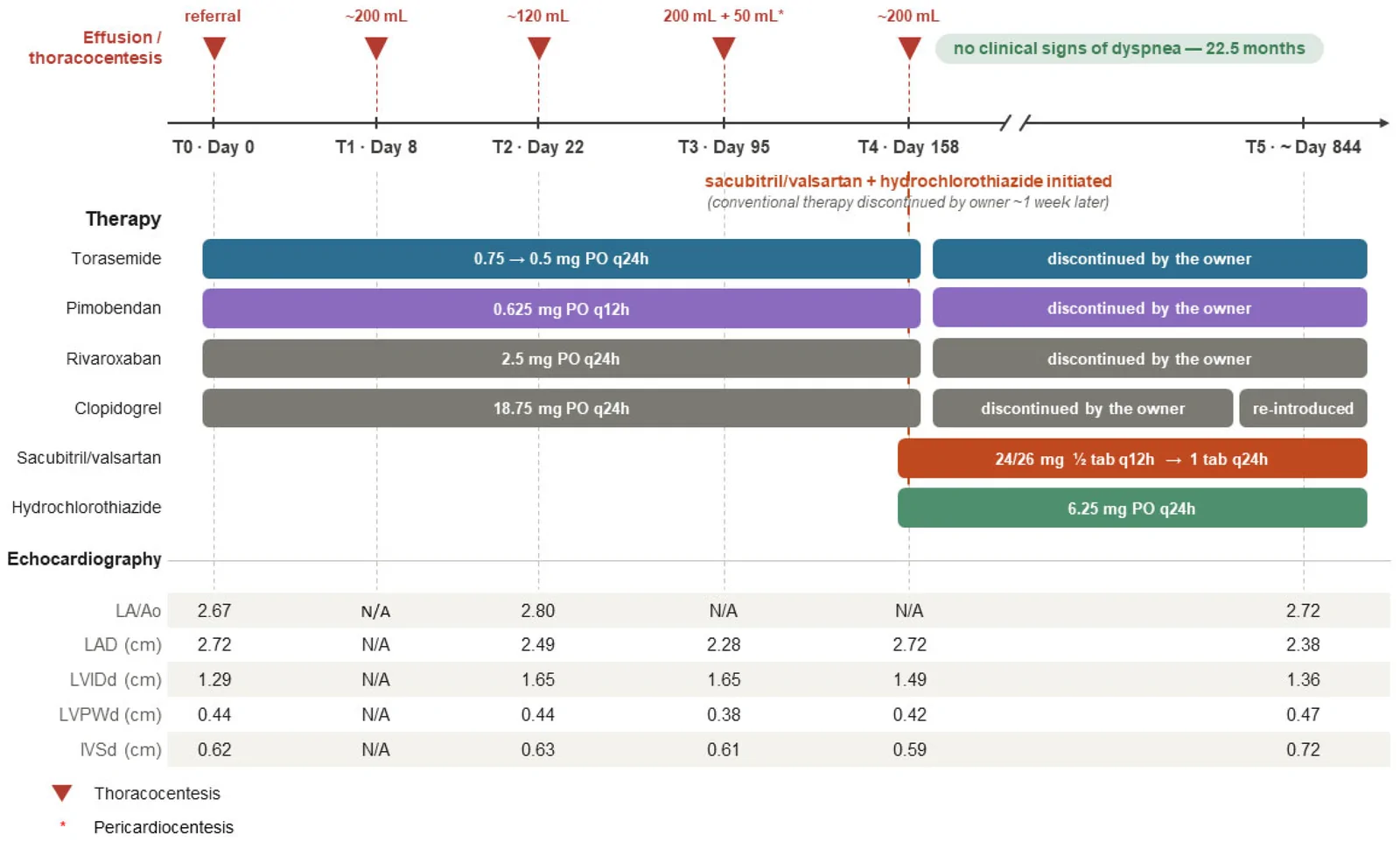
Schematic representation of the basic echocardiographic measurements and therapy adjustments at different timepoints during the follow-up of a cat with recurrent cardiogenic pleural effusion. Abbreviations: LA/Ao—left atria to aorta ratio, LAD—left atrium antero-posterior diameter, LVIDd—left ventricular internal diameter in diastole, LVPWd—left ventricular posterior wall in diastole, IVSd—interventricular septum in diastole, N/A—not available.

Echocardiographic re-evaluation was performed approximately two years after treatment initiation, and the findings are presented in Table 1. No pleural or pericardial effusion was identified. Severe biatrial enlargement and spontaneous echocardiographic contrast persisted. Although LAD decreased from 27.2 mm to 23.8 mm, the LA/Ao ratio increased slightly (2.67 to 2.72) and right atrial diameter increased from 12.9 mm to 16.6 mm. Atrial fibrillation remained present. Given the persistent spontaneous echocardiographic contrast and atrial fibrillation, clopidogrel therapy was reintroduced. Left ventricular fractional shortening increased from 36.4% to 50.7%. Follow-up hematologic and biochemical testing was declined by the owner because the cat remained clinically stable and free of overt clinical signs. The basic echocardiographic measurements and therapy adjustments during cardiologic examinations are presented in Figure 4.

## 3. Discussion

This paper presents a case of a cat with recurrent CHF treated with sacubitril/valsartan and hydrochlorothiazide. To the best of the authors’ knowledge, this is the first report of successful remittance of CHF and a follow up after two years in a cat treated with sacubitril/valsartan and hydrochlorothiazide. Before introduction of sacubitril/valsartan and hydrochlorothiazide, the cat experienced multiple episodes of congestive heart failure characterized by repeated pleural effusion requiring thoracocentesis. Despite treatment with pimobendan, torasemide, and antithrombotic therapy, recurrent decompensation occurred over several months.

Over the course of roughly two years after sacubitril/valsartan and hydrochlorothiazide were administered, there were no more relapses of clinical signs associated with CHF. No additional thoracocentesis procedures were required, and the owner reported sustained clinical stability without changes in respiratory rate or pattern. According to recommendations proposed by Ware, the use of sacubitril/valsartan in dogs and cats with refractory congestive heart failure appears promising, with a suggested dosage range of 5–10 mg/kg administered every 12 to 24 h. The dosage used in the present case was selected within this proposed therapeutic range [17].

An unexpected aspect of this case was that, approximately one week after release, the owner changed the recommended treatment schedule on their own initiative and without consulting a veterinarian. According to the owner, long-term treatment was subsequently maintained primarily with sacubitril/valsartan and hydrochlorothiazide.

Hydrochlorothiazide was added as part of a sequential nephron blockade strategy because recurrent congestive episodes persisted despite loop diuretic therapy and may have value in patients that develop resistance to furosemide. Although clinical evidence regarding hydrochlorothiazide administration in cats with CHF remains limited, thiazide diuretics have been recommended as adjunctive therapy in cases of refractory congestion and suspected diuretic resistance [16].

A clinically relevant observation was that sustained remission persisted even after owner-directed discontinuation of torasemide, leaving sacubitril/valsartan and hydrochlorothiazide as the sole ongoing medications. The individual contributions of these two agents cannot be separated retrospectively. In human medicine, valsartan and hydrochlorothiazide demonstrate a synergistic antihypertensive effect through complementary mechanisms, RAAS blockade and distal nephron natriuresis, with no clinically relevant pharmacokinetic interaction and a favorable tolerability profile [20]. Hydrochlorothiazide alone, however, would not be expected to sustain remission in a cat with such advanced structural disease over a two-year period. A more likely contributor to the prolonged absence of pleural effusion is the neurohormonal modulation achieved through ARNI. Persistent RAAS activation drives sodium retention, myocardial fibrosis, and adverse remodeling in feline heart failure; sacubitril/valsartan addresses this through simultaneous RAAS suppression and natriuretic peptide augmentation, an effect that neither agent achieves alone. Improvements in left ventricular systolic indices were observed at long-term follow-up, with fractional shortening increasing from 36.4% to 50.7%. Similar improvements in systolic performance and reverse remodeling have been reported following sacubitril/valsartan administration in human patients with heart failure and in experimental canine models of cardiorenal syndrome [13,21]. These effects are believed to result from attenuation of maladaptive neurohormonal activation, reduction in myocardial fibrosis [22], and favorable ventricular remodeling rather than from a direct positive inotropic action of the drug. Experimental canine studies have demonstrated that sacubitril/valsartan significantly reduces aldosterone exposure while also increasing natriuretic peptide activity, which leads to better natriuresis and decreased maladaptive neurohormonal activation [15]. Whether a similar interaction contributed to the clinical response observed in this case remains unknown. Therefore, it may be hypothesized that improved long-term control of RAAS activation may have contributed to the absence of recurrent pleural effusion in the present case. Although this mechanism is biologically plausible and supported by experimental canine studies, it remains speculative in the present case because no direct measurements of RAAS activation were obtained.

The initial regimen included pimobendan, prescribed in view of the reduced systolic function and nonspecific cardiomyopathic phenotype and supported by evidence of a survival benefit in cats with congestive heart failure secondary to cardiomyopathy [23]. This indication remains debated, as a prospective randomized study failed to confirm such a benefit and reported worsening of dynamic left ventricular outflow tract obstruction in a minority of treated cats [24,25]. As it was among the medications the owner discontinued during remission, its individual role cannot be established, and its withdrawal was not followed by an apparent recurrence of clinical decompensation. However systolic anterior motion of the mitral valve was associated with only mild dynamic left ventricular outflow tract obstruction.

Furthermore, follow-up echocardiography revealed complete resolution of both pleural and pericardial effusions, mild reductions in left atrial dimensions, and improved left ventricular systolic indices, while severe atrial enlargement and spontaneous echocardiographic contrast persisted. The increase in right atrial size observed at follow-up may have been related to persistent atrial fibrillation and progressive atrial remodeling. However, no signs of right-sided congestive heart failure developed during the observation period. Multiple left ventricular bands were identified in this cat; these are a common anatomical variant of the feline heart, present in virtually all examined feline hearts without morphological differences between normal and diseased specimens [26], while a higher prevalence within the outflow tract has been reported in obstructive hypertrophic cardiomyopathy [27,28]. Left ventricular bands have been associated with ventricular arrhythmias and repolarization abnormalities in human medicine [29], and one morphology canine study revealed the presence of Purkinje fibers within these structures [30]. The cat in this study showed no ventricular arrhythmia during any of the exams; however, left ventricular bands may contribute to a diastolic dysfunction leading to LA enlargement [31]. In the present case, the severe left atrial enlargement and advanced atrial remodeling provide a more plausible substrate for atrial fibrillation; however the implication of left ventricular bands in the diastolic dysfunction leading to atrial enlargement cannot be excluded. Taken together, these findings do not support complete reverse cardiac remodeling; they suggest substantial long-term stabilization of congestive heart failure despite persistence of advanced structural cardiac disease. It is worth noting that the cat is alive and free of clinical signs at the time of writing, resulting in a period of 25.5 months.

Several limitations should be acknowledged. First, this report describes a single clinical case, and therefore no causal relationship can be established between the combination of sacubitril/valsartan and hydrochlorothiazide administration and the favorable long-term outcome observed. Second, the treatment protocol was modified by the owner without veterinary supervision, resulting in discontinuation of loop-diuretic therapy and maintenance of only sacubitril/valsartan and hydrochlorothiazide; consequently, the individual contribution of each medication to the observed clinical response cannot be determined. Third, follow-up hematologic and biochemical analyses were not available because additional testing was declined by the owner, limiting evaluation of long-term renal function and electrolyte status. Lastly, systemic arterial blood pressure was not measured at presentation or during the follow-up period, precluding assessment of the hypotensive effects of the ARNI-thiazide combination and preventing exclusion of systemic hypertension as a potential contributor to the observed cardiomyopathic phenotype. Furthermore, serum total thyroxine (T4) concentration was not measured, and hyperthyroidism was therefore not formally excluded.

## 4. Conclusions

This study describes the long-term outcome of a cat with congestive heart failure treated with sacubitril/valsartan and hydrochlorothiazide. The two-year absence of dyspnea recurrence in a patient with severe structural disease and prior repeated decompensations is clinically noteworthy. Whether this outcome reflects a specific effect of ARNI therapy, a contribution of concomitant hydrochlorothiazide, or both, cannot be determined from a single observation. Prospective dose-finding and safety studies in cats with refractory CHF are warranted before broader clinical recommendations can be made.

## Figures and Tables

**Table 1:** Echocardiographic variables obtained during initial presentation and last examination, approximately two years after sacubitril/valsartan and hydrochlorothiazide initiation.

Echocardiographic Parameter	Initial Presentation	Last Examination
IVSd max (basal interventricular septum)	0.62 cm	0.73 cm
LVPWd max	0.44 cm	0.47 cm
LVIDd	1.29 cm	1.36 cm
LVIDs	0.82 cm	0.67 cm
LAD	2.72 cm	2.38 cm
LA/Ao	2.67	2.72
RAD	1.29 cm	1.66 cm
LV FS%	36.43%	50.7%
SEC	Present	Present
E wave velocity	109.53 cm/s	68.51 cm/s
TR vel	228.18 cm/s (20.8 mmHg)	242.54 cm/s (23.5 mmHg)
LVOT peak velocity	2.4 (23 mmHg)	1.8 m/s (13 mmHg)
Pleural effusion	Present	Absent
Pericardial effusion	Mild	Absent

IVSd = interventricular septal thickness in diastole; LVPWd = left ventricular free wall thickness in diastole; LVIDd = left ventricular internal diameter in diastole; LVIDs = left ventricular internal diameter in systole; LAD = left atrial antero-posterior diameter; LA/Ao = left atrial-to-aortic root ratio; RAD = right atrium diameter in diastole; LV FS% = left ventricular fractional shortening; SEC = spontaneous echocardiographic contrast; TR = tricuspid regurgitation; LVOT = left ventricular outflow.

## Data Availability

The original contributions presented in this study are included in the article. Further inquiries can be directed to the corresponding author.

## Abbreviations

The following abbreviations are used in this manuscript:

ARNI	Angiotensin receptor–neprilysin inhibitor
CHF	Congestive heart failure
HCM	Hypertrophic cardiomyopathy
RCM	Restrictive cardiomyopathy
RAAS	Renin–angiotensin–aldosterone system
IVSd	Interventricular septal thickness in diastole
LVPWd	Left ventricular free wall thickness in diastole
LVIDd	Left ventricular internal diameter in diastole
LVIDs	Left ventricular internal diameter in systole
LA/Ao	Left atrial-to-aortic root ratio
RAD	Right atrial diameter
FS	Fractional shortening
SEC	Spontaneous echocardiographic contrast
LVOT	Left ventricular outflow tract
TR	Tricuspid regurgitation
